# Epidemiology and outcomes of hospitalized adults with respiratory syncytial virus: A 6‐year retrospective study

**DOI:** 10.1111/irv.12643

**Published:** 2019-04-11

**Authors:** Henry Schmidt, Arighno Das, Hannah Nam, Amy Yang, Michael G. Ison

**Affiliations:** ^1^ Florida Atlantic University Schmidt College of Medicine Boca Raton Florida; ^2^ Northwestern University Feinberg School of Medicine Chicago Illinois

**Keywords:** hospitalized, mortality, pneumonia, ribavirin, RSV

## Abstract

**Objectives:**

Respiratory syncytial virus (RSV) is an important cause of morbidity and mortality in adults. Existing studies are limited by the number of seasons studied and most have focused on the immunocompromised.

**Methods:**

A retrospective cohort study was conducted on all adults (≥18 years) with a positive RSV molecular test admitted from 2009 to 2015 to one hospital in Chicago, IL. Epidemiologic and outcomes data were collected after IRB approval.

**Results:**

Of the 489 eligible patients, 227 had RSV A and 262 had RSV B. Patients had a median age of 61 years and comorbidity (eg, chronic lung disease [40.6%], obesity [37.8%], and cardiac disease [34.3%]). On presentation, most had cough (86.5%), fever (42.4%), and shortness of breath (38.2%). Severe disease was present in 27.6% of patients. Antibiotic was used in 76.3% inpatients and 45.8% at discharged despite few patients (4.7%) having documented bacterial infections. Supplemental oxygen and mechanical ventilation were utilized in 44.6% and 12.3%, respectively, while ICU level care was required in 26.9%. Most patients were discharged home (82.7%). Most deaths (68.4%, 13/19) were attributed to pneumonia or hypoxemia likely from RSV. Most fatal cases were seen in those with recent cancer treatment and older adults.

**Conclusions:**

Respiratory syncytial virus in hospitalized adults is associated with significant morbidity and mortality with 26.9% requiring ICU level care. Antibiotics are commonly prescribed to patients with documented RSV, and antibiotics are frequently continued after diagnosis. Novel antiviral therapies are needed for RSV to improve outcomes and potentially improve antibiotic stewardship in patients without a bacterial infection.

## 1 INTRODUCTION

Respiratory syncytial virus (RSV) is a single‐stranded RNA virus that is well recognized as a cause of significant morbidity and mortality among children. Its impact on adults has been increasingly recognized with the wider use of molecular testing of adult patients presenting with respiratory tract infections. It has been estimated that RSV infects 3%‐10% of adults annually.^1^ RSV typically presents in adults as self‐limited upper and lower respiratory tract infections. More severe presentations range from exacerbation of underlying pulmonary disease (asthma and chronic obstructive pulmonary disorder [COPD]) to severe lower respiratory tract infections such as pneumonia.^2^ RSV has been estimated to cause 177 525 excess hospitalizations each year and 14 000 excess deaths, in the United States.^1^, ^3^ Risk factors for more severe disease include old age (>65 years), solid organ (especially lung) transplant, hematopoietic stem cell transplant, and those with underlying lung disease.^4^ Among nursing home residents, RSV causes significant numbers of hospitalizations and deaths each winter.^5^ One study found that 12.5% of elderly patients hospitalized for an influenza‐like illness had RSV detected.^6^

Unlike influenza, there are no vaccines or antivirals approved for the prevention or treatment of RSV in adults. Palivizumab, a humanized monoclonal antibody against the RSV F glycoprotein, is approved for the prevention of RSV in select infants and children but has not been approved in adults as it has not been widely studied.^1^, ^7^ Several live attenuated and sub‐unit RSV vaccines are in advanced stages of development.^6^, ^8^ Aerosolized ribavirin is approved for the treatment of hospitalized infants and young children while aerosolized, oral, and intravenous ribavirin has been used selectively in immunocompromised adults with RSV with variable results.^9^, ^10^ RSV‐containing antibody preparations (monoclonal or intravenous immunoglobulins) have also been used in conjunction with ribavirin.^11^ Currently, two small molecule drugs, presatovir (GS‐5806, a fusion inhibitor) and lumicitabine (ALS‐008176, a polymerase inhibitor), are undergoing phase 2 studies in hospitalized and immunocompromised adults (ClinicalTrials.gov Identifiers: NCT02135614, NCT02254421, and NCT02254408).^12^, ^13^, ^14^ In addition, several other RSV‐active antivirals are in late pre‐clinical and phase 1 development and hold promise for the prevention and treatment of RSV.^15^

The burden of RSV infections in immunocompromised adults that are non‐HSCT is not well known.^16^ Most epidemiologic studies of RSV in hospitalized adults have been retrospective single‐center studies covering a single or few seasons.^2^, ^17^, ^18^ This retrospective cohort study was conducted to better define the epidemiology and outcomes of RSV in hospitalized adults over multiple seasons.

## 2 METHODS

### 2.1 Patient identification

After IRB approval, a retrospective cohort study on adults hospitalized with an RSV infection was conducted. All patients admitted to either of the participating hospitals at our institution (Northwestern Memorial Hospital and Prentice Women's Hospital) were included if they were 18 years of age or older and admitted from April 1, 2009, to March 31, 2015, with a positive RSV molecular test from nasopharyngeal specimens, bronchoalveolar lavage (BAL) samples, and sputum samples (xTag Luminex RVP [Luminex, Toronto, ON, Canada], GenMark Dx eSensor RVP [GenMark, Carlsbad, CA, USA], Pro‐Flu+ [Hologic, Marlborough, MA, USA], and Simplexa Flu A/B & RSV [Focus, Cypress, CA, USA]). Patients admitted only to the emergency room or ED observation unit were excluded from the study.

A small number of patients were admitted more than once during these six seasons, and if they had a repeat positive RSV molecular test, only unique admissions were included. It was thought that subsequent positive results on subsequent admissions could potentially represent prolonged viral shedding rather than new infection.^19^

### 2.2 Data collection

The Northwestern Medicine Enterprise Data Warehouse (EDW) was utilized to identify eligible patients and to capture key data from the electronic health record. Manual chart review was then conducted independently by two of the authors to abstract supplementary data available within the free text in the medical records (ie, presenting symptoms). Clinical data collected included demographics, comorbidities, onset of symptoms, presenting symptoms, use of supplemental oxygen requirement, ventilator support, need for renal replacement, antimicrobials, and bronchodilators, as well as duration of hospitalization, discharge location, and vital status on discharge.

### 2.3 Definitions

Each season was defined as May 1 through April 30 of the next year. Severe illness was defined as a patient who was given non‐invasive positive‐pressure ventilation (NIPPV), intubated, or admitted to the ICU. The presence of abnormal imaging was defined as the presence of pulmonary infiltrates described by a radiologist on a chest X‐ray or computed tomography (CT) scan of the chest. Nosocomial RSV infection defined as cases where symptoms began while a patient was in a hospital or other healthcare facility for ≥72 hours prior to a positive test for RSV.

### 2.4 Statistical analysis

Simple descriptive statistics (frequency and percent for categorical variables; mean and standard deviation or median and range for continuous variables) summarized sample demographic characteristics and clinical measures. Analyses employed Pearson's chi‐squared test to assess the association between severe disease status and symptoms, and between chronic disease and death, discharge status, ICU admission, and intubation status.

Analyses further utilized Fisher's exact method when expected cell counts were less than five for a given cross‐tabulation. All analyses assumed a 5% level of significance. We did not adjust for multiple hypothesis testing since the study is exploratory in nature and controlling for type II error rate is of more importance than type I error rate. Analyses were performed using R: A language and environment for statistical computing (R Foundation for Statistical Computing, Vienna, Austria; version 3.3.2).

## 3 RESULTS

### 3.1 Demographics

During the 6‐year study period, a total of 489 hospitalized RSV cases were identified: 55 in 2009‐2010; 54 in 2010‐2011; 46 in 2011‐2012; 55 in 2012‐2013; 115 in 2013‐2014; and 164 in 2014‐2015. During this same period of time, 283 557 unique individuals were admitted to the hospital with approximately 47 200 admissions each year. During this period, 16 449 (5.8%) of the patients had respiratory virus testing performed; rates of performed test increased over the study period with the largest increases the last two seasons. Of the hospitalized RSV‐infected patients, 46% (n = 227) had RSV A and 54% (n = 262) had RSV B; 60.5% were female and the mean age was 60 years (SD 17; see Table 1). Most (93.3%) RSV‐infected patients had a history of at least one medical comorbidity, while the most common comorbidities were chronic lung disease (40.6%), obesity (BMI ≥ 30, 37.8%), and cardiac disease (34.3%). Over a third of patients were taking an immunosuppressive medication (39.6%) or had a history of smoking (39.6%). While lung disease was the most common comorbidity, obstructive lung disease (83.5%) made up a majority of these patients, followed by other chronic lung illnesses (eg, active or prior lung cancer, pulmonary embolism, or abscess) (10.7%), restrictive lung disease (4.6%), and mixed obstructive/restrictive (1.5%).

**Table 1:** Demographics of hospitalized adults

Demographic	2009‐2010				2010‐2011				2011‐2012				2012‐2013				2013‐2014				2014‐2015				Total
	RSV A (%)		RSV B (%)		RSV A (%)		RSV B (%)		RSV A (%)		RSV B (%)		RSV A (%)		RSV B (%)		RSV A (%)		RSV B (%)		RSV A (%)		RSV B (%)		
N	45 (82)	10 (18)		11 (20)		43 (80)		22 (48)		24 (52)		37 (67)		18 (33)		34 (30)		81 (70)		78 (48)		86 (52)		489	
Age (mean)	63.3	62.2		58.4		56.6		56.3		58.8		57.9		60.1		57.4		61.3		62.9		60.2			
18‐59	16 (36)	3 (30)		6 (55)		23 (53)		12 (55)		13 (54)		18 (49)		8 (44)		17(50)		38 (47)		32 (41)		40 (46)		226	
60‐75	15 (33)	4 (40)		4 (36)		13 (30)		7 (32)		7 (29)		13 (35)		8 (44)		11 (32)		26 (32)		23 (29)		26 (30)		157	
>76	14 (31)	3 (30)		1 (9)		7 (16)		3 (14)		4 (17)		6 (16)		2 (11)		6 (18)		17 (21)		23 (29)		21 (24)		107	
Male	17 (38)	4 (40)		5 (45		22 (51)		7 (32)		15 (63)		11 (30)		8 (44)		16 (47)		36 (44)		28 (36)		25 (29)		194	
BMI																									
<20	10 (22)	1 (10)		0 (0)		2 (5)		3 (14)		2 (8)		1 (3)		1 (6)		3 (9)		7 (9)		8 (10)		8 (9)		46	
20‐24.99	10 (22)	0 (0)		3 (27)		17 (40)		8 (36)		7 (29)		11 (30)		4 (22)		8 (24)		19 (23)		14 (18)		21 (24)		122	
25‐29.99	13 (29)	3 (30)		2 (18)		14 (33)		2 (9)		6 (25)		4 (11)		6 (33)		8 (24)		22 (27)		22 (28)		23 (26)		125	
30‐34.99	3 (7)	3 (30)		1 (9)		4 (9)		5 (23)		7 (29)		8 (22)		3 (17)		5 (15)		9 (11)		12 (15)		13 (15)		73	
35‐39.99	1 (2)	2 (20)		1 (9)		0 (0)		2 (9)		0 (0)		4 (11)		1 (6)		3 (9)		8 (10)		8 (10)		13 (15)		43	
>40	6 (13)	0 (0)		3 (27)		5 (12)		2 (9)		1 (4)		8 (22)		3 (17)		6 (18)		9 (11)		12 (15)		7 (8)		62	
Nosocomial RSV	5 (11)	1 (10)		1 (9)		2 (5)		0 (0)		3 (13)		3 (8)		1 (6)		1 (3)		8 (10)		4 (5)		5 (6)		34	
Underlying medical conditions																									
Lung disease	16 (36)	4 (40)		7 (64)		14 (33)		8 (36)		8 (33)		12(32)		9 (50)		17 (50)		32 (40)		34 (44)		38 (44)		199	
Smoking	9 (20)	6 (60)		6 (55)		22 (51)		7 (32)		13 (54)		11(30)		6 (33)		11 (32)		35 (43)		29 (37)		39 (45)		194	
Chemotherapy <30 d	8 (18)	1 (10)		2 (18)		10 (23)		6 (27)		4 (17)		7 (19)		2 (11)		3 (9)		16 (20)		9 (12)		15 (17)		83	
SCT <1 y	3 (7)	0 (0)		0 (0)		5 (12)		3 (14)		2 (8)		3 (8)		0 (0)		1 (3)		5 (6)		3 (4)		16 (18)		41	
Diabetes	14 (31)	3 (30)		4 (36)		9 (21)		7 (32)		6 (25)		9 (24)		7 (39)		11 (32)		18 (22)		25 (32)		20 (23)		133	
Cardiac disease	16 (36)	3 (30)		2 (18)		12 (28)		10 (45)		6 (25)		10 (27)		8 (44)		7 (21)		28 (35)		36 (46)		30 (34)		168	
Rheum disease	7 (16)	0 (0)		2 (18)		10 (23)		3 (14)		7 (29)		4 (11)		3 (17)		6 (18)		13 (16)		11 (14)		13 (15)		79	
Renal disease	8 (18)	0 (0)		0 (0)		8 (19)		4 (18)		5 (21)		9 (24)		3 (17)		11 (32)		21 (26)		20 (26)		18 (21)		107	
SOT	4 (9)	0 (0)		1 (9)		4 (9)		2 (9)		0 (0)		1 (3)		4 (22)		3 (9)		3 (4)		2 (3)		6 (7)		30	
Immunosuppression	18 (40)	5 (50)		7 (64)		14 (33)		7 (32)		10 (42)		12 (32)		7 (39)		12 (35)		29 (36)		28 (36)		37 (43)		186	
Abnormal CXR	21 (47)	4 (40)		6 (55)		14 (33)		8 (41)		10 (42)		14 (38)		5 (28)		11 (32)		28 (35)		33 (42)		35 (40)		190	
Presenting symptoms																									
Cough	40 (89)	7 (70)		10 (91)		39 (91)		20 (91)		20 (83)		28 (76)		16 (89)		30 (88)		72 (89)		68 (87)		74 (85)		424	
Fevers	24 (53)	9 (90)		1 (9)		23 (53)		13 (59)		9 (38)		16 (43)		10 (56)		14 (41)		29 (36)		28 (36)		32 (37)		208	
Shortness of breath	19 (42)	4 (40)		5 (45)		17 (40)		11 (50)		11 (46)		16 (43)		7 (39)		9 (26)		32 (40)		32 (41)		24 (28)		187	
Nasal congestion	11 (24)	3 (30)		0 (0)		17 (40)		7 (32)		5 (21)		7 (19)		5 (28)		1 (3)		18 (22)		19 (24)		22 (25)		115	
Rhinorrhea	6 (13)	2 (20)		2 (18)		8 (19)		5 (23)		5 (21)		1 (3)		5 (28)		5 (15)		19 (23)		17 (22)		22 (25)		97	
Chills	9 (20)	1 (10)		0 (0)		10 (23)		4 (18)		7 (29)		4 (11)		2 (11)		5 (15)		15 (19)		14 (18)		16 (18)		87	
Sore throat	13 (29)	3 (30)		0 (0)		7 (16)		3 (14)		5 (21)		4 (11)		3 (17)		3 (9)		12 (15)		14 (18)		12 (14)		79	
Fatigue	9 (20)	2 (20)		0 (0)		8 (19)		4 (18)		1 (4)		5 (14)		1 (6)		4 (12)		10 (12)		10 (13)		13 (15)		67	
Myalgias	4 (9)	2 (20)		2 (18)		12 (28)		4 (18)		4 (17)		8 (22)		1 (6)		4 (12)		10 (12)		8 (10)		7 (8)		66	
Headache	2 (4)	1 (10)		2 (18)		3 (7)		0 (0)		3 (13)		5 (14)		1 (6)		3 (9)		4 (5)		11 (14)		9 (10)		44	

### 3.2 Clinical presentation

The most common symptom on presentation was cough (86.5%), fever (42.4%), shortness of breath (38.2%), nasal congestion (23.5%), and rhinorrhea (19.7%). Chest imaging on admission had documentation of pulmonary infiltrate in 190 patients (38.8%). The median time between onset of symptoms and admission was 4.8 days for all patients and 3.3 days for patients who died (*P* = 0.20). The median length of time between symptom onset and +RVP test was 4.9 days for all patients and 7.8 days for patients who died (*P* = 0.16).

### 3.3 Management and outcomes

Supplemental oxygen was utilized in 44.6% (218) patients with an additional 11.9% (58) requiring non‐invasive ventilation, such as CPAP, and 12.3% (60) requiring mechanical ventilation at some point during their hospitalization (see Table 2). Most (76.3%, 374) patients were administered at least one dose of antibiotics during their hospitalization. 45.8% (213) patients were discharged on antibiotics. There were documented co‐infections based on positive cultures, PCR, or urine antigens in 40 (8.2%): of these, 23 (4.7%) were bacterial (11 pneumonia, 10 UTI, and seven bacteremia), 16 (3.3%) were viral (five rhinovirus, five influenza, and six other [HSV, VZV, coxsackie, and norovirus]), and three (0.6%) were fungal (one Aspergillus pneumonia, one Candida UTI, and one Candida esophageal) co‐infections; some patients had multiple co‐infections. While chest imaging data were not collected, pulmonary infections were only documented in 12 (2.5%) patients and three patients with bacteremia likely had bacteremia secondary to pneumonia.

**Table 2:** Treatment and outcomes of hospitalized adults

	2009‐2010		2010‐2011		2011‐2012		2012‐2013		2013‐2014		2014‐2015		Total
	RSV A (%)	RSV B (%)	RSV A (%)	RSV B (%)	RSV A (%)	RSV B (%)	RSV A (%)	RSV B (%)	RSV A (%)	RSV B (%)	RSV A (%)	RSV B (%)	
Antibiotic use	41 (91)	8 (80)	9 (82)	38 (88)	21 (95)	18 (75	28 (76	13 (72	21 (62)	54 (67)	43 (55)	52 (60)	374
Documented co‐infection	4 (9)	1 (10)	2 (18)	6 (14)	8 (36)	2 (8)	8 (22)	2 (11)	8 (24)	14 (17)	10 (13)	7 (8)	72
Renal replacement	4 (9)	0 (0)	0 (0)	2 (5)	2 (9)	5 (21)	5 (14)	1 (6)	3 (9)	6 (7)	1 (1)	5 (6)	34
Bronchodilator use	29 (64)	6 (60)	8 (73)	30 (70)	20 (91)	18 (75)	26 (70)	13 (72)	22 (65)	53 (65)	47 (60)	48 (56)	320
Supplemental O_2_	23 (51)	6 (60)	7 (64)	20 (47)	14 (64)	13 (54)	21 (57)	10 (56)	11 (32)	32 (40)	28 (36)	34 (40)	218
ICU Admission	17 (38)	1 (10)	5 (45)	12 (28)	8 (36)	8 (33)	11 (30)	6 (33)	8 (24)	19 (23)	19 (24)	18 (21)	132
NIPPV	7 (16)	0 (0)	4 (36)	7 (16)	5 (23)	3 (13)	6 (16)	4 (22)	3 (9)	8 (10)	8 (9)	3 (3)	58
Intubation	7 (16)	0 (0)	4 (37)	6 (14)	4 (18)	7 (29)	5 (14)	4 (22)	4 (12)	8 (10)	8 (10)	5 (6)	60
Outcome													
Discharge home	35 (78)	9 (90)	7 (64)	35 (81)	17 (77)	19 (79)	30 (81)	14 (78)	29 (85)	66 (81)	65 (83)	79 (92)	405
Other medical facility	8 (18)	1 (10)	4 (36)	6 (14)	2 (9)	3 (13)	6 (16)	4 (22)	2 (6)	13 (16)	7 (9)	7 (8)	63
Death	2 (4)	0 (0)	0 (0)	2 (5)	3 (14)	2 (8)	1 (3)	0 (0)	2 (6)	2 (2)	4 (5)	1 (1)	19

Severe disease was documented in 27.6% (135) of patients. The median length of stay in the ICU was 2.9 days (range 1‐78 days; six patients had ICU admissions ≥30 days) and the median number of days of mechanical ventilation was six (range 1‐77 days). Oncology and hematopoietic stem cell transplant recipients had a lower rate of ICU admission and hospitalization compared to the general population (see Table S2).

Most patients (82.7%, 405) were discharged home after a median hospital length of stay of 3.6 days (range 1‐53 days). An additional 63 patients (12.9%) were discharged to other medical facilities such as rehabilitation centers or skilled nursing facilities, after a median length of stay of 11.6 (range 1‐74) days. Nineteen (3.9%) patients died after a median length of stay of 16 days (range 2‐82) days. Of the deaths, 13 (68.4%) were attributed to pneumonia or hypoxemia likely from RSV. The cause of death of the other six patients included cardiac arrest, aggressive oncologic disease, and sepsis from other sources. Most (73.7%, 14/19) were in the ICU at the time of death (see Table S1 for details). Oncology and hematopoietic stem cell transplant recipients had a higher mortality rate than the general population (see Table S2).

### 3.4 Risk factors for outcomes

Shortness of breath on presentation (52.6% vs 33.3%, *P* < 0.001) was associated with severe disease while cough (91.5% vs 76.3%, *P* < 0.001), rhinorrhea (23.4% vs 10.4%, *P* = 0.002), nasal congestion (27.7% vs 11.1%, *P* < 0.001), and myalgias (15.8% vs 7.4% *P* = 0.022) on presentation were less likely to develop severe disease (see Table 3). No pre‐existing condition was significantly over‐represented in patients requiring ICU admission, although the presence of a co‐infection (26.5% vs 10.6%, *P* < 0.001) was more common among ICU admissions. No factor was statistically associated with intubation, although there was a trend toward higher rates of intubation in patients with cancer treatment, diabetes, and a documented co‐infection (see Table 4).

**Table 3:** Correlation between 347 patients with severe disease and presenting signs and symptoms

Sign/Symptom	Symptom present with severe disease, N = 135 (%)	Symptom present without severe disease, N = 354 (%)	*P* value
Cough	103 (76.3)	324 (91.5)	<0.001
Nasal congestion	16 (11.9)	98 (27.7)	<0.001
Rhinorrhea	14 (10.4)	83 (23.4)	0.002
Sore throat	15 (11.1)	64 (18.1)	0.083
Headache	9 (6.7)	35 (9.9)	0.349
Fevers	54 (40.0)	156 (44.1)	0.478
Chills	22 (16.3)	66 (18.6)	0.637
Myalgias	10 (7.4)	56 (15.8)	0.022
Shortness of breath	71 (52.6)	118 (33.3)	<0.001
Fatigue	14 (10.4)	53 (15.0)	0.24
Arthralgias	0 (0.0)	1 (0.3)	0.999

**Table 4:** Outcomes by chronic disease or condition

	N	Pregnant (%)	Lung disease (%)	Recent cancer treatment (%)	HSCT (%)	Diabetes (%)	Cardiac disease (%)	Rheumatologic disease (%)	CKD (%)	SOT (%)	Co‐infection (%)	Age >65 (%)	Obesity (%)	Morbid obesity (%)
ICU admission	132	0	61 (46.2)	19 (14.4)	7 (5.3)	40 (30.3)	55 (41.7)	25 (18.9)	30 (22.7)	11 (8.3)	35 (26.5)	57 (43.2)	50 (37.9)	22 (16.7)
No ICU admission	357	8 (2.2)	138 (38.7)	65 (18.2)	33 (9.2)	93 (26.1)	117 (32.8)	54 (15.1)	78 (21.8)	19 (5.3)	38 (10.6)	145 (40.6)	128 (37.5)	40 (11.7)
*P* value		*0.115*	*0.16*	*0.391*	*0.22*	*0.41*	*0.085*	*0.38*	*0.932*	*0.308*	<0.001	*0.683*	*0.937*	*0.181*
Intubated	60	60	0 ( 0.0)	29 (48.3)	6 (10.0)	2 (3.3)	20 (33.3)	24 (40.0)	12 (20.0)	11 (18.3)	4 (6.7)	17 (28.3)	24 (40.0)	23 (39.0)
Not intubated	429	429	8 (1.9)	170 (39.6)	78 (18.2)	38 (8.9)	113 (26.3)	148 (34.5)	67 (15.6)	97 (22.6)	26 (6.1)	56 (13.1)	178 (41.5)	155 (37.6)
*P* value			0.604	0.252	0.164	0.207	0.324	0.489	0.499	0.561	0.776	0.004	0.936	0.954
Died	19	0	5 (26.3)	8 (42.1)	2 (10.5)	7 (36.8)	5 (26.3)	4 (21.1)	6 (31.6)	0	5 (26.3)	13 (68.4)	7 (36.8)	1 (5.3)
Alive	470	8 (1.7)	194 (41.3)	76 (16.2)	38 (8.1)	126 (26.8)	167 (35.5)	75 (16)	102 (21.7)	30 (6.4)	68 (14.5)	189 (40.2)	171 (37.8)	61 (13.5)
*P* Value		*1*	*0.238*	0.009	*0.663*	*0.484*	*0.472*	*0.527*	*0.462*	*0.621*	*0.182*	0.027	*0.999*	*0.491*
Home	407	8 (2.0)	163 (40.0)	68 (16.7)	35 (8.6)	103 (25.3)	134 (32.9)	64 (15.7)	90 (22.1)	24 (5.9)	52 (12.8)	152 (37.3)	148 (36.4)	49 (12.0)
D/C to outside healthcare facility	63	0	31 (49.2)	8 (12.7)	3 (4.8)	23 (36.5)	33 (52.4)	11 (17.5)	12 (19.0)	6 (9.5)	16 (25.4)	37 (58.7)	23 (36.5)	12 (19.0)
*P* value		*0.605*	*0.216*	*0.535*	*0.455*	*0.086*	0.004	*0.869*	*0.7*	*0.413*	0.014	0.002	*0.999*	*0.21*

CKD, any diagnosed stage of chronic kidney disease or end‐stage renal disease; Co‐infection, any concurrently documented infection; HSCT, hematopoietic stem cell transplant within the past year; Morbid obesity, BMI ≥ 40; Obesity, BMI ≥30; SOT, any history of solid organ transplant (eg, kidney, liver).

Age >65 (58.7% vs 37.3%, *P* = 0.002), the presence of chronic cardiac disease (52.4% vs 32.9%, *P* = 0.004), and the presence of co‐infection (25.4% vs 12.8%, *P* = 0.014) were associated with discharge to a skilled nursing of rehabilitation facility (see Table 4). Fatal cases occurred more frequently in patients with recent cancer treatment (42.1% vs 16.2%, *P* = 0.009) and patients with age >65 (68.4% vs 40.2%, *P* = 0.027).

## 4 DISCUSSION

This study represents one of the largest studies of RSV in hospitalized adults with patients sampled over multiple seasons. About a quarter of patients required ICU level care, and death was more common among those with recent cancer treatment and age >65 years. Most patients were given antibacterial therapy during hospitalization with almost half being discharged on antibiotics despite limited documentation of concomitant bacterial infection. These data provide important insight into the epidemiology and outcomes of RSV in hospitalized adults in the contemporary era.

The inclusion of six seasons worth of data provides insight into the seasonal variability of RSV in hospitalized adults. There was no significant variation in demographics, treatment, or outcomes by season or predominating RSV type. While most previous studies have found annual alternation between RSV A and RSV B, our data suggest a 3‐year cycle to be common as well (A predominant, B predominant, mixed‐type seasons).^20^, ^21^, ^22^, ^23^ Although RSV circulation patterns tend to be local, future studies are needed to define the degree of regional variability of predominating type. Most patients were documented in the typical winter respiratory virus months (November‐April). Such data will be helpful in the design of clinical studies and for assessments of the impact of future vaccine or treatments for RSV.

The finding that nasal congestion, rhinorrhea, cough, and myalgias were associated with a lack of severe disease may help identify patients who may require less supportive measures and can be discharged more quickly. Likewise, the association between older age, prior cardiac disease, and co‐infection and need for higher level of care on discharge likewise may help risk stratify patients on admission. While these correlations with symptoms are interesting, lack of prospective symptom collection may have missed symptoms in the sicker population (ie, patients in the ICU may not have sore throat or rhinorrhea noted).

In comparison with other studies, this study suggests similar data regarding symptoms at presentation, median length of stay, and rates of co‐infection, antimicrobial therapy, and death. The patients in our study were somewhat younger than other studies that included hospitalized adults as well.^17^, ^18^ However, our study suggests that rates of ICU admission and mechanical ventilation were higher overall than what is previously described.^17^ The relatively high proportion of immunocompromised patients included in this study may, in part, explain this high rate of ICU admission and ventilator use. The time from symptom onset to presentation was also similar to what has been documented in other studies.^18^

Additionally, the finding that ICU and intubation rates were lower among patients with recent chemotherapy or HSCT compared to healthier patients is interesting. While death rates were higher in the immunocompromised patients, other risk factors such as underlying lung disease may play a larger role in respiratory decompensation. It is also possible that the immunocompromising conditions result in less inflammation which could be protective against the need for ICU care or intubation. Intubation is associated with higher mortality in immunocompromised patients, and the lower rate may be due to avoidance of invasive procedures in these populations.^24^ The lower death rate among healthy individuals is otherwise unremarkable given the inherent physiologic reserve these patients may have to rebound from illness.

An interesting finding is that patients who died had a longer period of time between symptom onset and diagnostic testing. The reason for this is not entirely obvious with the data collected in this study. The likely factor that was difficult to obtain in the retrospective analysis was the clinical decision making of the providers. Many of the fatal cases occurred in patients who were immunosuppressed or recently received chemotherapy which may make symptoms less severe. Further, nosocomial acquisition of infection is also a possibility. This may be likely because the length of stay prior to ICU transfer was relatively long in many of the fatal cases. As such, non‐specific symptoms such as fever may not trigger respiratory viral testing in all patients. Future prospective studies may be able to confirm this finding and better understand drivers of fatal cases.

As this study was observational in nature, there are a few limitations to be considered. A significant fraction of the data was obtained via retrospective chart review, especially subjective data including presenting symptoms. As such, undocumented symptoms cannot be assessed potentially adding bias. While discharge location is easily identified in the chart review, it was not always apparent if a patient resided in a long‐term care facility prior to admission. This may overstate the impact of RSV on the added costs of discharge location being anywhere other than home. There is likely also a significant testing bias as it is protocol to test all ICU patients on admission with a respiratory virus panel and to test all immunocompromised patients with fever or respiratory symptoms. Testing of other populations is more at the discretion of the admitting physician.^25^ Lastly, as this is a single‐center study, unique features of our center or location may affect the findings of this study and future studies in other regions are needed to confirm our findings.

Another finding of this study is that despite the availability of rapid molecular diagnostic methods, empiric antibacterial therapy was prescribed to a significant portion of RSV‐positive patients despite lack of documented bacterial co‐infection. This finding is congruent with other studies that demonstrate similar findings.^26^ While molecular diagnostic tests may aid in the detection of respiratory viruses such as RSV or influenza, the true impact of viral diagnostics on clinical practice still remains unclear.

While this study generally confirmed many of the findings of older studies, it is one of the largest studies using contemporary molecular diagnostics on a large population of RSV‐infected hospitalized adults. As such, it provides insight that will become increasingly important as a diagnosis of RSV may soon have therapeutic implications with many such agents actively undergoing study.^13^, ^14^ RSV remains a significant cause of morbidity in adults, leading to high rates of ICU admission, mechanical ventilation, and death. Most patients received at least a single dose of antibiotics without a source of bacterial infection documented suggesting an opportunity for antibiotic stewardship. The use of rapid RSV molecular detection should be further embedded into larger antibiotic stewardship efforts. While this effort alone would not be sufficient to reduce antibiotic overuse, it may serve a valuable role if implemented alongside potential serum biomarkers and/or educational interventions.

## CONFLICT OF INTEREST

HS, HN, AD, and AY have no conflicts of interest to declare. MGI has received research support, paid to Northwestern University, from Beckman Coulter, Cepheid, Chimerix, Gilead, Janssen, and Shire; he has received payment as a consultant from Celltrion, Chimerix, Farmark, Genentech/Roche, Toyama/MediVector, Seqirus, and Shionogi; he has provided unpaid consultation to BioCryst, Cellex, GlaxoSmithKline, NexBio, Romark, Unither Virology, and Vertex; and he is a paid member of data and safety monitoring boards for GlaxoSmithKline and Shionogi.
