# Supplementary material for: Epidemiology and outcomes of hospitalized adults with respiratory syncytial virus: A 6‐year retrospective study
Source: Influenza Other Respir Viruses. 2019 Apr 11;13(4):331–8. doi: 10.1111/irv.12643 (PMC6586178; doi:10.1111/irv.12643)
Supplement: Supplementary file 1 [file IRV-13-331-s001.docx]

**Supplemental Table 1. Characteristic of Deceased Patients**

| Sex | RSV  Type | Age | Hospital | ICU | Chronic Illness | APACHE | SOFA | O2 | Bronchodilator | Intubation | Dialysis | Listed Cause(s) of Death | Respiratory COD |
| --- | --- | --- | --- | --- | --- | --- | --- | --- | --- | --- | --- | --- | --- |
| F | A | 71 | 43 | 13 | Lung Disease, Diabetes, Co-infection |  |  | X | X |  |  | pneumonia, hypoxic respiratory failure, clostridium difficile, MRSA bacteremia, acute kidney injury | X |
| M | A | 70 | 16 | 6 | Recent Chemo |  |  | X | X | X |  | pneumonia, hypoxic respiratory failure, acute kidney injury | X |
| M | B | 61 | 37 | 9 | Recent Chemo, CKD |  |  | X | X |  | X | sepsis, cardiac death |  |
| F | B | 31 | 83 | 78 | Rheumatologic Dz, Co-infection |  |  | X | X | X |  | Acute Myelogenous Leukemia, clostridium difficile |  |
| M | B | 84 | 6 | 1 | Rheumatologic Dz, CKD |  |  | X | X | X |  | NSTEMI, Heart Failure, Acute on Chronic Renal Failure |  |
| F | A | 42 | 5 | 2 | Lung Disease, Recent Chemo, Diabetes, Co-infection | 16 | 5 | X | X | X |  | hypoxic respiratory failure | X |
| M | A | 75 | 11 | 9 | Recent Chemo, Cardiac Dz | 22 |  | X | X |  |  | pneumonia, hypoxic respiratory failure | X |
| M | B | 83 | 9 | 3 | Cardiac Dz, Rheumatologic Dz, CKD | 50 | 13 | X | X | X | X | pneumonia, hypoxic respiratory failure, sepsis, cardiac arrest | X |
| F | A | 43 | 13 | 7 | Lung Disease, Recent Chemo, Diabetes | 10 |  | X | X | X |  | hypoxic respiratory failure, widespread metastatic disease | X |
| F | A | 73 | 20 | 7 | Recent Chemo, Co-infection | 26 | 12 | X |  |  |  | Mantle Cell Lymphoma and Sepsis |  |
| M | A | 56 | 30 | 8 | Lung Disease, CKD, Co-infection | 32 | 12 | X | X | X |  | pneumonia, bronchiectasis, ruptured esophageal varices | X |
| M | B | 69 | 4 | 1 | Diabetes, CKD |  |  |  | X |  | X | pneumonia | X |
| M | B | 66 | 26 | 0 | Recent Chemo, Diabetes, Cardiac Dz |  |  | X | X |  |  | relapsed multiple myeloma, DIC, MSOF |  |
| M | A | 42 | 21 | 1 | Co-infection |  |  | X |  | X |  | hypoxic respiratory failure | X |
| F | A | 83 | 9 | 3 | None documented |  |  | X | X |  |  | hypoxic respiratory failure, cecal volvulus, Alzheimer's Dementia | X |
| M | A | 74 | 39 | 13 | Lung Disease, Cardiac Dz, Rheumatologic Dz, Co-infection |  |  | X | X | X |  | acute heart failure, stroke, mediastinitis, pneumonia, sepsis |  |
| M | B | 72 | 20 | 1 | Recent Chemo, Diabetes, Co-infection |  |  | X | X |  |  | pneumonia, cardiac arrest | X |
| F | A | 85 | 2 | 0 | Cardiac Dz, CKD |  |  |  |  |  | X | pneumonia, cardiac arrest | X |
| F | A | 87 | 11 | 9 | Diabetes |  |  | X | X | X |  | pneumonia, hypoxic respiratory failure | X |

**Definitions:** Hospital – days hospitalized; ICU – days spent in ICU; CKD - any diagnosed stage of chronic kidney disease or end-stage renal disease; Co-infection – any concurrently documented infection; APACHE – calculated APACHE II score; SOFA – calculated Sequential Organ Failure Assessment score; O2 – use of supplemental oxygen; COD – cause of death; MSOF – multi-system organ failure

**Supplemental Table 2. Demographics and Outcomes by At-Risk Population**

|  | n | Age | Most Common Co-Morbidities | Intubated | ICU admission | Died | D/C Home |
| --- | --- | --- | --- | --- | --- | --- | --- |
| HSCT | 40 | 56  (27-77) | 1. Immunosuppression Meds 78%  2. Chemo 68% 3. Lung & CKD 18% | 2 (5%) | 7 (17.5%) | 2 (5%) | 35 (87.5%) |
| Recent Chemo | 84 | 62  (26-84) | 1. Immunosuppression Meds 52%  2. HSCT 32% 3. Lung 27% | 6 (7.1%) | 19 (22.6%) | 8 (9.5%) | 68 (81.0%) |
| Neither | 392 | 61  (18-102) | 1. Lung 44% 2. Cardiac 40%  3. Immunosuppression Meds 33% | 53 (13.5%) | 110 (28.1%) | 11 (2.8%) | 324 (82.7%) |
| Nosocomial RSV | 78 | 65  (23-97) | 1. Immunosuppression Meds 53%  2. Lung 33% 3. Cardiac & Chemo 33% | 19 (24.3%) | 34 (43.6%) | 7 (8.9%) | 50 (64.1%) |
